# Evaluation of DNA yield from various tissue and sampling sources for use in single nucleotide polymorphism panels

**DOI:** 10.1038/s41598-024-56128-9

**Published:** 2024-05-17

**Authors:** David L. Pearce, Jessie E. Edson, Chris S. Jennelle, W. David Walter

**Affiliations:** 1 Pennsylvania Cooperative Fish and Wildlife Research Unit, The Pennsylvania State University, 413 Forest Resources Building, University Park, PA 16802 USA; 2 Minnesota Department of Natural Resources, 5463 West Broadway Ave., Forest Lake, MN 55025 USA; 3 U.S. Geological Survey, Pennsylvania Cooperative Fish and Wildlife Research Unit, The Pennsylvania State University, 403 Forest Resources Building, University Park, PA 16802 USA; 4 Present Address: Department of Rangeland, Wildlife and Fisheries Management, Texas A&M University, College Station, TX 77843 USA; 5 Present Address: Minnesota Department of Natural Resources, Division of Ecological and Water Resources, Nongame Wildlife Program, St Paul, MN 55155 USA

**Keywords:** Population genetics, Sequencing

## Abstract

Genetics studies are used by wildlife managers and researchers to gain inference into a population of a species of interest. To gain these insights, microsatellites have been the primary method; however, there currently is a shift from microsatellites to single nucleotide polymorphisms (SNPs). With the different DNA requirements between microsatellites and SNPs, an investigation into which samples can provide adequate DNA yield is warranted. Using samples that were collected from previous genetic projects from regions in the USA from 2014 to 2021, we investigated the DNA yield of eight sample categories to gain insights into which provided adequate DNA to be used in ddRADseq or already developed high- or medium-density SNP panels. We found seven sample categories that met the DNA requirements for use in all three panels, and one sample category that did not meet any of the three panels requirements; however, DNA integrity was highly variable and not all sample categories that met panel DNA requirements could be considered high quality DNA. Additionally, we used linear random-effects models to determine which covariates would have the greatest influence on DNA yield. We determined that all covariates (tissue type, storage method, preservative, DNA quality, time until DNA extraction and time after DNA extraction) could influence DNA yield.

## Introduction

Genetic studies have been used by wildlife managers and researchers to understand population structure and diversity^1^, individual fitness^2^, impacts of habitat fragmentation^3^, and the potential spread of disease^4^. Microsatellites have been the primary method for genetic studies, however, a transition is underway to using single nucleotide polymorphisms (SNPs) to identify individuals and make inferences at a population level^5,6^. Comparison studies between SNPs and microsatellites indicated that SNPs provide considerably more information for population analysis^7,8^ and that SNPs perform as well or outperform microsatellites in determining population structure, genetic diversity, and genetic differentiation^5,6,9^. While microsatellites can be sufficient at determining population structure, even among closely related populations^10^, they are not as efficient as SNPs in determining genetic diversity or relatedness^11–13^. This is due to the ability of generating and filtering thousands of SNPs with relative ease^14^ resulting in a larger quantity of loci sampled compared to microsatellites^6,9,15^. Increasing the number of loci sampled per individual enables a precise measure of a population’s genetic structure and diversity, thus increasing the effectiveness of genetic studies and making SNPs a powerful tool for wildlife conservation and management.

Microsatellites require considerably less DNA than SNPs, requiring samples concentrated to 10–20 ng/μL of DNA to use 1 μL of DNA in a 10 μL polymerase chain reaction (PCR)^16,17^. A frequently used SNP sequencing method for wildlife genetic studies, double digest restricted site associated DNA sequencing (ddRADSeq) requires a minimum DNA concentration of 20 ng/μL at a volume of 5–6 μL^18,19^, and can produce ~ 18,907–35,099 SNPs for a population-level analysis^20,21^. A SNP sequencing panel specific to white-tailed deer (*Odocoileus virginianus)* offers two arrays, a high-density and a medium-density array^22^. The high-density array can produce ~ 600,000 SNPs and requires a minimum DNA concentration of 10 ng/μL and a volume of 50μL per sample^22^. The medium-density array can produce ~ 60,000 SNPs, requiring a minimum DNA concentration of 17.2 ng/μL and a volume of 25μL per sample^22^. Use of different SNP sequencing methods is largely limited by the types of samples that are used and the yield of quality DNA that they produce.

Genetic laboratories are often tasked with explaining the best method for collecting samples for a study species even though detailed resources are not readily available on tissue type, collection method, storage, and preservative used to maintain adequate quantities of DNA, but see^17,23^. The common approaches to collecting DNA are invasive and non-invasive sampling. The difference in these two approaches is invasive sampling involves taking a sample from the animal which can be either destructive or non-destructive to the animal and non-invasive sampling refers to collecting a sample without handling the animal. Samples collected using non-invasive techniques (e.g., hair or feces) often result in low DNA yields^24–26^. Invasive samples produce higher DNA yields and can be collected from subjects obtained by hunter harvest, vehicle mortalities or capture events. These samples can include blood, retropharyngeal lymph nodes, buccal swabs, muscle, organ, ear, and skin tissues that can be collected either destructively or non-destructively from a subject animal^17,21,27–31^. Moreover, sample handling is a crucial component to studies and proper handling can reduce DNA degradation from UV light, heat, and improper storage^32–34^.

Due to the DNA concentration and volume requirements for SNP sequencing and the factors that can contribute to DNA degradation, a thorough investigation into which samples are best suited for genetics studies using SNPs is warranted. Our study assessed the yield of quality DNA from various tissue samples that were collected invasively using destructive and non-destructive methods for white-tailed deer. We assume that samples collected from white-tailed deer and DNA yield from samples can serve as a proxy for other mammalian species. Our objectives were to assess the factors that could influence DNA yield and we hypothesized that components of sample collection (e.g., storage, preservative) would influence DNA yield across sample categories. This information will be a valuable resource for wildlife researchers and managers looking to use SNPs to ask management and conservation questions about wildlife populations.

## Materials and methods

### Sample collection

We sampled tissue from white-tailed deer as part of studies from New York, Pennsylvania, and Minnesota, USA from 2014 to 2021 during disease surveillance, genetic analysis, and capture and marking efforts^17,31^. All samples underwent microsatellite analysis and/or sanger sequencing and were obtained during chronic wasting disease (CWD) surveillance and management efforts by state wildlife agency personnel or approved by The Pennsylvania State University IACUC (PROTO201800026, #46,581) and all methods were carried out in accordance with relevant guidelines and regulations. All samples provided through hunter harvest were obtained within 1–3 days of harvest, and thus carcass condition did not influence DNA yield prior to our collection of tissue described below.

We excised muscle from carcasses provided by hunters and stored muscle frozen at − 20 °C in whirl-paks (a sterile sampling bag, [Nasco Sampling LLC, Pleasant Prairie, WI, USA]) with no preservative for six months. We then subsampled approximately a 1 mm x 3 mm x 5 mm slice of neck muscle, taped it to a piece of card stock, and allowed it to air-dry at room temperature before shipping to the laboratory in coin envelopes. We collected ear tissue from harvested deer punched into AllFlex tubes (ET-All) with an applicator (Valley Vet Supply, Marysville KS, USA) stored in a patented preservative at room temperature for two months until DNA extraction. The patented preservative was a combination of ultrapure water, sodium chloride, tris hydrochloride, disodium EDTA and additional components with composition available by request (FertiPro N.V, Belgium). We also collected ear tissue from live deer during collaring efforts that were stored in 90% ethanol (ET-Eth) and frozen at − 20 °C for two years until DNA extraction (The Pennsylvania State University IACUC PROTO201800026). We collected retropharyngeal lymph nodes (RLNs) that were frozen immediately at − 20 °C with no preservative for ten months prior to DNA extraction. We also collected RLNs that were refrigerated with no preservative for three weeks prior to DNA extraction. We collected sections of tongue excised from carcasses of hunter harvested deer then stored in 90% ethanol at − 20 °C for five months until DNA extraction. We collected blood from captive white-tailed deer stored in Vacuette 6 ml K2E EDTA K2 tubes (K2-EDTA, [Greiner Bio-One, Monrow, NC, USA]) and frozen at − 20 °C for up to four weeks until DNA extraction (The Pennsylvania State University IACUC #46,581). We collected nasal mucosa using sterile cotton-tipped applicators from hunter harvest carcasses, placed in a sterile vacutainer tube with no preservative, and frozen for 1 year at − 20 °C before DNA extraction.

### DNA extractions

All samples used in this analysis have been stored at − 20 °C since DNA extraction. All samples were extracted using QIAGEN Extraction Kits (QIAGEN, Valencia, CA, USA) and approximately four mm square of tissue (ear, muscle, tongue and lymph nodes) was excised to standardized DNA extractions. QIAGEN DNeasy tissue extraction protocol was followed with no modifications for frozen RLNs and one modification of eluting with 150μL AE buffer in the final step for tongue, refrigerated RLNs and ET-Eth. The QIAGEN DNeasy blood extraction protocol was followed for blood samples using 200 mL of blood for extraction. The QIAGEN DNeasy tissue extraction protocol was followed for the nasal mucosa samples by taking approximately 20% of the cotton swab and digesting it in QIAGEN ATL buffer and proteinase K solution, followed by a centrifugation step, to spin down the cotton swab and transfer the supernatant to a new microcentrifuge tube before following the remaining steps with a final elution step of 150μL to assist in concentrating DNA. The QIAGEN QIAamp tissue extraction protocol was performed on a QIaCube HT (QIAGEN, Valencia, CA, USA) automated extractor for ET-All and dried muscle tissue samples. All samples were digested for a minimum of three hours and up to 24 h at 56 °C.

### Laboratory analysis

We used a Nanodrop Lite spectrophotometer (quality measure, [ThermoFisher Scientific, Waltham, MA, USA]) and a Qubit 4 fluorometer (quantity measure, [ThermoFisher Scientific, Waltham, MA, USA]) for measurements of extracted DNA quantity in ng/μL and quality using 260/280. We used a Qubit due to the increased accuracy of estimating the true yield of DNA in a sample due to the fluorescent dye in the assay that binds to double stranded DNA (dsDNA)^35^. The protocol for quantity measures followed the Biotium AccuGreen dsDNA Broad Range (BR) Assay protocol where 1μL of eluted DNA was added to 199μL of the dsDNA BR Assay (ThermoFisher Scientific, 2015). DNA quality measures used 1μL of eluted DNA placed onto the Nanodrop pedestal.

We assessed DNA degradation by using a Genomic DNA ScreenTape on an Agilent TapeStation 4150 (2024 Agilent Technologies, Inc) with 1–2 μL of DNA completed by the Penn State Huck Institutes of Life Sciences. Samples that were over 300 ng/μL were diluted to 250 ng/μL to be used with the Genomic DNA ScreenTape. The facility determined the fragment size of the DNA in base pairs (bp) and the DNA Integrity Number (DIN) of the samples. The Agilent TapeStation provides a closer look at the level of DNA degradation of our samples using DIN, where samples are scored on a scale of 1–10, with higher values indicating intact DNA and lower values indicating degradation^36^.

### Statistical analysis

We conducted all analyses of sample DNA yield using R^37^ v4.2.0 in RStudio^38^ v 2022.02.4 + 500.pro1; R code and data used in this analysis are available on the USGS repository^39,40^. We calculated mean, median and standard deviation with *psych*^41^ using the describe() function. Additionally, we generated boxplots using *ggplot2*^42^ where the quantity for each tissue sample is categorized by storage or preservative. We calculated the number of samples (n = 20) for each sample category that met the requirements for SNP panels (hereafter panel). The panel requirements were standardized to ng/μL and samples that met requirements for ddRADseq, high-density and medium-density panels were given a 1 and samples that did not meet the requirements were given a 0.

We fit linear mixed-effects models using the *lme4*^43^ after selecting 5 covariates that have been considered to influence DNA yield from previous research^32–34^ including: 1) tissue – blood, ear, RLN, muscle, nasal mucosa, and tongue; 2) preservative – Allflex, dried, ethanol, K2-EDTA, and none; 3) storage – frozen (− 20 °C), refrigerated, and room temperature; 4) pre-extraction – time (in days) in storage until DNA extraction; 5) post-extraction – time (in days) in storage after DNA extraction. The quality of DNA was treated as a random effect for all models and Akaike’s Information Criterion for small sample sizes (AICc) was used for model selection using the aictab() function from *AICcmodavg*^44^.

## Results

**Figure 1:**
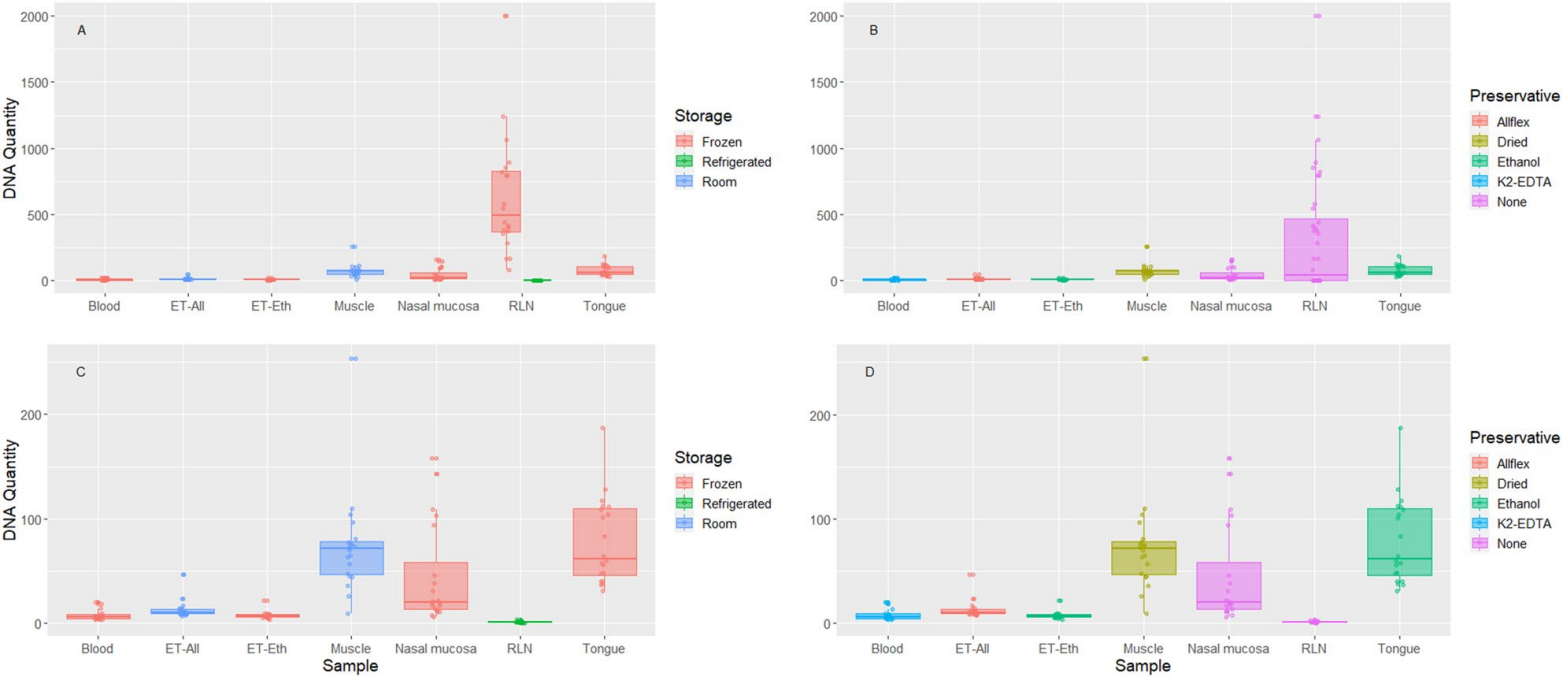
Boxplots of DNA quantity (ng/μL) for tissue sample category collected (dried muscle, ear stored in Allflex [ET-All] or ethanol [ET-Eth], frozen retropharyngeal lymph nodes [RLNs], refrigerated RLNs, tongue, blood, and nasal mucosa). Evaluated tissue categories were sorted by (**A**) storage method, (**B**) preservative (**C**) storage method without frozen RLNs and (**D**) preservative without frozen RLNs.

There was a vast amount of variation in DNA yield among sample categories (Fig. 1) and seven categories met requirements for ddRADseq, high-density and medium-density panels (Table 1). Frozen RLNs had a median quantity of 495 ng/μL (Table 2, and SI Table 1) and had 20 samples that met requirements for all panels. Dried muscle had 19 samples that met requirements for all panels with a median quantity of 71.3 ng/μL. Tongue and nasal mucosa also met requirements for all panels, with a median quantity of 61.7 ng/μL and 20.2 ng/μL, respectively. Tongue had 20 samples that met ddRADseq requirements and high- and medium-density panels requirements. Nasal mucosa had 10 samples that met ddRADseq requirements, with 18 samples for high- and 12 for medium-density panels. ET-All, ET-Eth and blood also met requirements for all panels and had a median quantity of 10.01 ng/μL, 6.51 ng/μL, 6.04 ng/μL, respectively. ET-All had two samples that met ddRADseq requirements; and 10 samples met requirements for the high-density panel and two samples for the medium-density panel. ET-Eth had one sample that met requirements for ddRADseq, high-, and medium density panels. Blood had two samples that met requirements for ddRADseq, four samples that met requirements for the high-density panel, and three samples for the medium-density panel. Refrigerated RLNs did not meet requirements for ddRADseq, high-density or medium-density panels and had a median quantity of 0.783 ng/μL.

**Table 1:** Number of samples (n = 20) from each tissue sample category (dried muscle, ear stored in Allflex [ET-All] or ethanol [ET-Eth], frozen retropharyngeal lymph nodes [RLNs], refrigerated RLNs, tongue, blood, and nasal mucosa) that met DNA quantity requirements for double digest restricted site associated DNA sequencing (ddRADSeq), high-, and medium-density single nucleotide polymorphism (SNP) panels.

SNP panel	DNA requirements (ng/μL)	Dried muscle	ET-All	ET-Eth	Frozen RLN	Refrigerated RLN	Tongue	Blood	Nasal mucosa
ddRADSeq	20	19	2	1	20	0	20	2	10
High-density	10	19	10	1	20	0	20	4	18
Medium-density	17.2	19	2	1	20	0	20	3	12

**Table 2:** Median values of DNA quantity (ng/μL), DNA quality (260/280), DNA Integrity Number (DIN), and Fragment Size (bp) and pre-extraction time (in days), preservative and storage method for each tissue sample category collected (dried muscle, ear stored in Allflex [ET-All] or ethanol [ET-Eth], frozen retropharyngeal lymph nodes [RLNs], refrigerated RLNs, tongue, blood, and nasal mucosa) for evaluation of DNA requirements for use in double digest restricted site associated DNA sequencing, high-, and medium-density single nucleotide polymorphisms panels.

	Pre-extraction	Preservative method	Storage method	ng/μL	260/280	DIN	bp
Muscle	180	Dried	Room	71.3	1.96	7.6	18,231
ET-All	60	Allflex	Room	10.01	1.71	8.8	49,233
ET-Eth	913	Ethanol	Frozen	6.51	1.91	7.1	31,445
Frozen RLN	285	None	Frozen	495	1.91	6.4	13,720
Refrigerated RLN	21	None	Refrigerated	0.783	2.17	6.3	14,030
Tongue	150	Ethanol	Frozen	61.7	2.08	6.4	17,562
Blood	395	K2-EDTA	Frozen	6.04	1.77	8.4	23,194
Nasal Mucosa	365	None	Frozen	20.2	1.97	3.4	10,329

Fragment analysis showed that a higher DNA yield did not always indicate a higher level of DNA integrity. Frozen RLNs had a median DIN of 6.4 and a median fragment size of 13,720 bp with a median quality of 1.91, while refrigerated lymph nodes had a median DIN of 6.3, a median fragment size of 14,030 bp and a median quality of 2.17 (Table 2, SI Table 2–4). The ET-All had the highest level of integrity with a median DIN of 8.8 and a median fragment size of 49,233 bp with a quality of 1.71; however, nasal mucosa had the lowest level of integrity with a median DIN of 3.4, a median fragment size of 10,329 bp and a quality of 1.97. The ET-Eth, blood and dried muscle also had higher values of integrity with a median DIN of 7.1, 8.4, and 7.6; a median fragment size of 31,445 bp, 23,194 bp and 18,231 bp; and a median quality of 1.91, 1.77, and 1.96, respectively. Tongue had a DIN value of 6.4 a median fragment size of 17,562 bp, and a median quality of 2.08.

Four models were supported with AICc weights of 0.25 (Table 3). Tissue and storage were present in each of the four top models. Preservative was present in three of the four top models and pre- and post-extraction were present in two of the four top models.

**Table 3:** Table of linear mixed-effects models with number of parameters (K), Akaike’s Information Criterion adjusted for small sample size (AICc), change in IAC (ΔAICc), and AiCc weight (AICcWt).

Model	K	AICc	ΔAICc	AICcWt
Tissue + preservative + storage + pre-extraction + post-extraction + (quality)	10	2022.34	0	0.25
Tissue + storage + pre-extraction + post-extraction + (quality)	10	2022.34	0	0.25
Tissue + preservative + storage + (quality)	10	2022.34	0	0.25
Tissue + storage + (quality)	10	2022.34	0	0.25
Tissue + preservative + pre-extraction + post-extraction + (quality)	10	2033.49	11.15	0.00
Tissue + pre-extraction + post-extraction + (quality)	10	2045.97	23.63	2E-06
Pre-extraction + post-extraction + preservative + storage + (quality)	10	2049.62	27.28	3E-07
Tissue + preservative + (quality)	9	2122.36	100.01	5E-23
Tissue + (quality)	8	2131.17	108.83	6E-25
(Quality)	3	2212.40	190.06	1E-42

Quality was included as a random effect in all models (quality).

## Discussion

We identified considerable variation in the yield of quality DNA across samples that are typically collected during research projects for mammals. Although all samples were collected and underwent microsatellites analysis and/or sanger sequencing^17,31^, not all samples met panel requirements and/or had high integrity. All frozen RLNs met requirements for all three panels but all refrigerated RLNs failed to meet panel requirements, making the case for proper handling and storage in the case of RLNs. Additionally, both RLNs sample categories had fragment sizes < 20,000 bp indicating that these samples may not be highly intact DNA^45^ which could be problematic in SNP sequencing. We recognize that restrictions may not allow for the collection of RLNs such as samples needed for CWD testing and thus not available for DNA. In these cases, dried muscle and tongue may be suitable destructive sampling alternatives. The majority of dried muscle and all tongue samples met DNA requirements for all three panels and had median fragment sizes close to 20,000 bp; however, there was considerable variation in DNA yield across samples. During DNA extractions we observed that samples from both categories that had higher DNA yields were predominantly muscle tissue and not adipose tissue or mucosa. If tongue or muscle samples are to be collected, samples should be comprised predominantly of muscle tissue.

We understand that destructive sampling may not always be possible if destruction of the animal is not desired. If collection efforts are restricted from sampling destructively and muscle samples are desired, samples collected using biopsies may be a suitable alternative^46–48^. Ear tissue offers an additional non-destructive alternative that is often collected during capture events, in which we assessed two categories: ET-All and ET-Eth. Each category had samples that met requirements for all three panels and had highly intact DNA with both categories having a median fragment size of > 30,000 bp. While ET-All had more samples that met requirements across all panels and a higher fragment size, we suspect the variability was due to samples having more muscle than hair or cartilage. We observed that samples with higher DNA yields for ET-All and ET-Eth were not just ear cartilage but had muscle tissue 1–2 mm thick. If ear tissue is collected, sampling from areas on the ear where tissue is the thickest appeared to yield the best results. If samples consist predominantly of cartilage, and results from that sample is still desired, DNA extraction from cartilage is possible^49^. While ear tissue categories yielded few samples with DNA yield sufficient to meet ddRadseq requirements, all tongue samples met ddRadseq requirements. Ear tissue and tongue have successfully been used in ddRadseq panels; using tongue and ear tissue collected from vehicle collisions, targeted sampling, and voluntary statewide CWD testing program, Chafin et al. (2021) was able to successfully use ddRadseq for the discernment of the population structure of white-tailed deer in Arkansas, USA^21^. It is unknown which samples from white-tailed deer yielded appropriate DNA for ddRadseq in Chafin et al. (2021) because the authors do not report on what method was used to collect what tissue and no evaluation of DNA yield per sample was reported as in our study.

Another non-destructive sample that is often collected during capture events is blood; which had a low number of samples that met panel requirements but a median fragment size of > 20,000 bp. With a low number of samples meeting panel requirements and challenges in collecting blood samples such as the ability to find a vein in a live animal under sedation^50^, blood may not be ideal. An alternative sample that can be easily collected is nasal mucosa. Although nasal mucosa in our study was collected from white-tailed deer post-mortem^17^, these samples can be collected during capture events^50^. At least half of the nasal mucosa samples met panel requirements but median fragment size was <20,000; if adjustments to the DNA extraction protocol were made, such as extracting more of the cotton swab or using a nonautomated ethanol-based technique^50^ DNA yield may increase.

We recognize that panels can have a total DNA requirement and may not be limited by a concentration of ng/μL, however, these requirements often involve a maximum volume; restricted by either PCR or tray size. With a fixed volume, the concentration of DNA influences which samples can be used for a panel and is the reason we standardized our analysis to ng/μL. Moreover, there are laboratory methods that can be used to increase the DNA concentration of a sample, and we used these methods for samples where DNA yield was a concern. We used methods such as eluting with a lower volume of elution buffer or double eluting to increase the concentration of DNA. There are other methods that can be used to increase the concentration of DNA, such as drying and resuspending extracted DNA or using a DNA concentration kit^51^; however, each of these techniques are still limited by the potential DNA yield of a sample.

Assessing the level of degradation using DIN identified some categories of tissue types that produced low quantities of DNA, such as ET-All and blood, but had high levels of integrity and would likely do well undergoing DNA concentration efforts. Samples with low DIN would likely not perform well regardless of concentration efforts due to high levels of fragmentation. It is worth considering what level of DNA degradation could impinge various genetic projects to help determine which sample types may be most suitable. A DIN number of seven for genome wide association studies^52^ and for next generation library preparation^53^ has been considered necessary; however, a study successfully used samples with a DIN under three for next generation sequencing^36^, and another used samples with a DIN of 1.3–6.2 for whole exome library preparation^54^.

Our models indicated that all covariates likely influence DNA yield, supporting the case for proper handling and storage of samples. Tissue type, preservative used, storage method, and time until DNA extraction were controlled by agency collaborators and limited our ability to standardize collection protocols for samples evaluated in our study; effectively reducing the inference that could have been made by the models. For example, there were two sampling protocols for RLNs and ear tissue, but other sample categories only had a single protocol. Additionally, the variation in covariates might not have been broad enough for all tissues to explain DNA yield. Future investigations into the influence of covariates on DNA yield may be helped if the methodology is standardized to one tissue type and that tissue’s potential DNA yield is known.

## Conclusion

Wildlife researchers from across the United States often inquire about appropriate protocols for collecting samples in population genetic studies of white-tailed deer. Our investigation into the yield of quality DNA revealed several categories met requirements for use in all three panels, providing options for researchers during field operations. It is important to emphasize how crucial proper handling of samples is in minimizing DNA degradation. We provide detailed results on sample DNA yield and integrity that will inform researchers on sample type, storage, and preservative to maximize their samples likelihood of being used in a panel of their choosing. There was no single sample type, storage approach, or preservative that excelled above others, but future studies that can include additional controls during sample collection may yield more definitive results.

### Supplementary Information

Supplementary Tables.

## Data Availability

Data is available at https://www.sciencebase.gov/catalog/item/64c133f9d34e70357a3293d8 and R code used in analysis is available at https://code.usgs.gov/cooperativeresearchunits/evaluation-of-dna-yield.

## References

[1] Proctor MF, McLellan BN, Strobeck C, Barclay RMR (2005). Genetic analysis reveals demographic fragmentation of grizzly bears yielding vulnerably small populations. Proc. R. Soc. B Biol. Sci..

[2] Wisely SM, Buskirk SW, Fleming MA, McDonald DB, Ostrander EA (2002). Genetic diversity and fitness in black-footed ferrets before and during a bottleneck. J. Hered..

[3] Keyghobadi N (2007). The genetic implications of habitat fragmentation for animals. Can. J. Zool..

[4] Miller WL, Walter WD (2020). Can genetic assignment tests provide insight on the influence of captive egression on the epizootiology of chronic wasting disease?. Evol. Appl..

[5] Smith CT (2007). Impacts of marker class bias relative to locus-specific variability on population inferences in chinook salmon: A comparison of single-nucleotide polymorphisms with short tandem repeats and allozymes. Trans. Am. Fish. Soc..

[6] Zimmerman SJ, Aldridge CL, Oyler-McCance SJ (2020). An empirical comparison of population genetic analyses using microsatellite and SNP data for a species of conservation concern. BMC Genom..

[7] Rosenberg NA, Li LM, Ward R, Pritchard JK (2003). Informativeness of genetic markers for inference of ancestry*. Am. J. Hum. Genet..

[8] Lao O, van Duijn K, Kersbergen P, de Knijff P, Kayser M (2006). Proportioning whole-genome single-nucleotide–polymorphism diversity for the identification of geographic population structure and genetic ancestry. Am. J. Hum. Genet..

[9] Liu N, Chen L, Wang S, Oh C, Zhao H (2005). Comparison of single-nucleotide polymorphisms and microsatellites in inference of population structure. BMC Genet..

[10] Lemopoulos A (2019). Comparing RADseq and microsatellites for estimating genetic diversity and relatedness—Implications for brown trout conservation. Ecol. Evol..

[11] Davey JW, Blaxter ML (2010). RADSeq: Next-generation population genetics. Brief. Funct. Genomics.

[12] Hauser L, Baird M, Hilborn R, Seeb LW, Seeb JE (2011). An empirical comparison of SNPs and microsatellites for parentage and kinship assignment in a wild sockeye salmon ( *Oncorhynchus nerka* ) population: Analytical approaches. Mol. Ecol. Resour..

[13] Thrasher DJ, Butcher BG, Campagna L, Webster MS, Lovette IJ (2018). Double-digest RAD sequencing outperforms microsatellite loci at assigning paternity and estimating relatedness: A proof of concept in a highly promiscuous bird. Mol. Ecol. Resour..

[14] Helyar SJ (2011). Application of SNPs for population genetics of nonmodel organisms: new opportunities and challenges: Analytical approaches. Mol. Ecol. Resour..

[15] Chen X, Sullivan PF (2003). Single nucleotide polymorphism genotyping: biochemistry, protocol, cost and throughput. Pharmacogenomics J..

[16] Bradbury IR (2015). Transatlantic secondary contact in Atlantic Salmon, comparing microsatellites, a single nucleotide polymorphism array and restriction-site associated DNA sequencing for the resolution of complex spatial structure. Mol. Ecol..

[17] Edson J, Brown J, Miller WL, Walter WD (2021). Comparison of sample types from white-tailed deer (Odocoileus virginianus) for DNA extraction and analyses. Sci. Rep..

[18] Payton, A. ddRADseq Protocol. (2015).

[19] Jordon-Thaden IE (2020). A basic dd RAD seq two-enzyme protocol performs well with herbarium and silica-dried tissues across four genera. Appl. Plant Sci..

[20] Peterson BK, Weber JN, Kay EH, Fisher HS, Hoekstra HE (2012). Double digest RADseq: An inexpensive method for de novo SNP discovery and genotyping in model and non-model species. PLoS ONE.

[21] Chafin TK (2021). Spatial population genetics in heavily managed species: Separating patterns of historical translocation from contemporary gene flow in white-tailed deer. Evol. Appl..

[22] Michigan Department of Natural Resources. White-tailed Deer Genetic Resources. https://storymaps.arcgis.com/stories/9794d395588b45d7a055e86bf42d602b (2022).

[23] *The Wildlife Techniques Manual*. (Johns Hopkins University Press, Baltimore, Md, 2012).

[24] Broquet T, Ménard N, Petit E (2006). Noninvasive population genetics: a review of sample source, diet, fragment length and microsatellite motif effects on amplification success and genotyping error rates. Conserv. Genet..

[25] Bunting S, Burnett E, Hunter RB, Field R, Hunter KL (2014). Incorporating molecular genetics into remote expedition fieldwork. Trop. Conserv. Sci..

[26] Poutanen J, Pusenius J, Wikström M, Brommer JE (2019). Estimating population density of the white-tailed deer in Finland using non-invasive genetic sampling and spatial capture-recapture. Ann. Zool. Fenn..

[27] DeYoung RW, Demarais S, Gonzales RA, Honeycutt RL, Gee KL (2002). Multiple paternity in white-tailed deer (*Odocoileus virginianus*) revealed by DNA microsatellites. J. Mammal..

[28] Lang KR, Blanchong JA (2012). Population genetic structure of white-tailed deer: Understanding risk of chronic wasting disease spread: White-tailed deer genetic structure and CWD. J. Wildl. Manag..

[29] Jansson E, Ruokonen M, Kojola I, Aspi J (2012). Rise and fall of a wolf population: Genetic diversity and structure during recovery, rapid expansion and drastic decline. Mol. Ecol..

[30] Budd K, Berkman LK, Anderson M, Koppelman J, Eggert LS (2018). Genetic structure and recovery of white-tailed deer in Missouri: Genetic structure of deer in Missouri. J. Wildl. Manag..

[31] Miller WL, Edson J, Pietrandrea P, Miller-Butterworth C, Walter WD (2019). Identification and evaluation of a core microsatellite panel for use in white-tailed deer (*Odocoileus virginianus*). BMC Genet..

[32] Vink CJ, Thomas SM, Paquin P, Hayashi CY, Hedin M (2005). The effects of preservatives and temperatures on arachnid DNA. Invertebr. Syst..

[33] Ballari RV, Martin A (2013). Assessment of DNA degradation induced by thermal and UV radiation processing: Implications for quantification of genetically modified organisms. Food Chem..

[34] Graham CF (2015). Impacts of degraded DNA on restriction enzyme associated DNA sequencing ( RADS eq). Mol. Ecol. Resour..

[35] Masago K (2021). Comparison between fluorimetry (Qubit) and spectrophotometry (NanoDrop) in the quantification of DNA and RNA extracted from frozen and FFPE tissues from lung cancer patients: A real-world use of genomic tests. Medicina.

[36] Hiramatsu K (2023). Diagnostic utility of DNA integrity number as an indicator of sufficient DNA quality in next-generation sequencing–based genomic profiling. Am. J. Clin. Pathol..

[37] R Core Team. *R: A Language and Environment for Statistical Computing*. R Foundation for Statistical Computing, Vienna, Austria (2021).

[38] RStudio Team. *RStudio: Integrated Development Environment for R*. RStudio, PBC, Boston, MA, URL http://www.rstudio.com/ (2020).

[39] Pearce, D. L. & Walter, W. D. Evaluation of DNA yield from various tissue and sampling sources for use in single nucleotide polymorphism panels. Version 1.0.0: U.S. Geological Survey software release. Reston, Va. 10.5066/P96QMZUE (2023).10.1038/s41598-024-56128-938760358

[40] Walter, W. D. & Pearce, D. Evaluation of DNA yield from various sources for use in single nucleotide polymorphism panels. U.S. Geological Survey data release, 10.5066/P96VPBSO (2023).

[41] Revelle, W. Procedures for Psychological, Psychometric and Personality Research. *R Package Version 233* (2023).

[42] Wickham, hadley *et al.* Create Elegant Data Visualisations Using the Grammer of Graphics. (2016).

[43] Bates, D. *et al.* Linear Mixed-Effects Models using ‘Eigen’ and S4. (2015).

[44] Mazerolle, M. J. Model Selection and multimodel Inference Based on (Q)AIC(c). (2023).

[45] Lucena-Aguilar G (2016). DNA source selection for downstream applications based on DNA quality indicators analysis. Biopreserv. Biobank..

[46] Karesh WB, Smith F, Frazier-Taylor H (1987). A remote method for obtaining skin biopsy samples. Conserv. Biol..

[47] Haley NJ (2016). Antemortem detection of chronic wasting disease prions in nasal brush collections and rectal biopsy specimens from white-tailed deer by real-time quaking-induced conversion. J. Clin. Microbiol..

[48] Mijele D (2016). A practical guideline to remote biopsy darting of wildebeests for genetic sampling. Int. J. Vet. Sci. Med..

[49] Lee, S. B. & Shewale, J. G. DNA Extraction Methods in Forensic Analysis. in *Encyclopedia of Analytical Chemistry* (ed. Meyers, R. A.) 1–18 (Wiley, 2017). 10.1002/9780470027318.a1104m.pub2.

[50] Neary MT (2014). Technical note: A comparison of DNA collection methods in cattle and yaks. J. Anim. Sci..

[51] Moore, D. & Dowhan, D. Purification and concentration of DNA from aqueous solutions. *Curr. Protoc. Mol. Biol.* **59**, (2002). 10.1002/0471142727.mb0201as59 18265306

[52] Herpe, Y.-E. *et al.* Quality control of genomic DNA for the French Kidney Disease Study by the Biobanque de Picardie. (2021).

[53] Diessl, N., Ernst, U., Schulz, A. & Wolf, S. Quality Control in illumina sequencing workflows using the tapestation system (2018).

[54] Elisa Viering *et al.* Implementation of automated sample quality control in whole exome sequencing. *J. Life Sci.* **11**, (2017).

