# Supplementary material for: Evaluation of DNA yield from various tissue and sampling sources for use in single nucleotide polymorphism panels
Source: Sci Rep. 2024 May 17;14:11340. doi: 10.1038/s41598-024-56128-9 (PMC11101418; doi:10.1038/s41598-024-56128-9)
Supplement: Supplementary file 1 — Supplementary Tables. [file 41598_2024_56128_MOESM1_ESM.docx]

**Supplementary Information**

Table 1: Descriptive statistics of DNA yield (ng/μL) and pre-extraction time (in days), preservative and storage method for each tissue sample category collected (dried muscle, ear stored in Allflex [ET-All] or ethanol [ET-Eth], frozen retropharyngeal lymph nodes [RLNs], refrigerated RLNs, tongue, blood, and nasal mucosa) for evaluation of DNA requirements for use in double digest restricted site associated DNA sequencing, high-, and medium-density single nucleotide polymorphisms panels.

| **Sample** | | **Min** | | **Max** | | **Mean** | | **Median** | | **StDev** | |
| --- | --- | --- | --- | --- | --- | --- | --- | --- | --- | --- | --- |
| Dried Muscle | | 8.82 | | 254 | | 73.94 | | 71.3 | | 49.31 | |
| ET-All | | 6.94 | | 46.2 | | 12.76 | | 10.01 | | 8.73 | |
| ET-Eth | 3.36 | | 21.8 | | 7.27 | | 6.51 | | 3.75 | |  |
| Frozen RLN | 82.6 | | 2000 | | 632.38 | | 495 | | 449.36 | |  |
| Refrigerated RLN | 0.208 | | 2.82 | | 0.99 | | 0.783 | | 0.73 | |  |
| Tongue | | 30.6 | | 187 | | 78.41 | | 61.7 | | 41.15 | |
| Blood | | 3.12 | | 20.2 | | 8.08 | | 6.04 | | 5.46 | |
| Nasal Mucosa | | 5.52 | | 158 | | 44.76 | | 20.2 | | 48.12 | |

Table 2: Descriptive statistics of DNA quality (260/280) for each tissue sample category collected (dried muscle, ear stored in Allflex [ET-All] or ethanol [ET-Eth], frozen retropharyngeal lymph nodes [RLNs], refrigerated RLNs, tongue, blood, and nasal mucosa) per 1μL of eluted DNA for evaluation of requirements for use in a double digest restricted site associated DNA sequencing, high-density, and medium-density sequencing panels.

| **Sample** | **Min** | **Max** | **Mean** | **Median** | **StDev** |
| --- | --- | --- | --- | --- | --- |
| Dried Muscle | 1.91 | 2.02 | 1.96 | 1.96 | 0.04 |
| ET-All | 1.38 | 2.05 | 1.71 | 1.71 | 0.16 |
| ET-Eth | 1.75 | 1.95 | 1.89 | 1.91 | 0.05 |
| Frozen RLN | 1.88 | 1.98 | 1.91 | 1.91 | 0.02 |
| Refrigerated RLN | 1.61 | 2.85 | 2.14 | 2.17 | 0.23 |
| Tongue | 1.88 | 2.11 | 2.06 | 2.08 | 0.06 |
| Blood | 0.91 | 2.42 | 1.69 | 1.77 | 0.34 |
| Nasal Mucosa | 1.9 | 2.16 | 2.00 | 1.97 | 0.09 |

Table 3: Descriptive statistics of DNA Integrity Number for each tissue sample category collected (dried muscle, ear stored in Allflex [ET-All] or ethanol [ET-Eth], frozen retropharyngeal lymph nodes [RLNs], refrigerated RLNs, tongue, blood, and nasal mucosa) per 1-2 μL of eluted DNA for evaluation of requirements for use in a double digest restricted site associated DNA sequencing, high-density, and medium-density sequencing panels.

| **Sample** | **Min** | **Max** | **Mean** | **Median** | **StDev** |
| --- | --- | --- | --- | --- | --- |
| Dried Muscle | 6.4 | 8.1 | 7.4 | 7.6 | 0.5 |
| ET-All | 7.8 | 9.4 | 8.7 | 8.8 | 0.5 |
| ET-Eth | 6.3 | 8.1 | 7.2 | 7.1 | 0.5 |
| Frozen RLN | 1.0 | 7.5 | 5.9 | 6.4 | 1.5 |
| Refrigerated RLN | 1.0 | 7.5 | 5.9 | 6.3 | 1.6 |
| Tongue | 4.3 | 7.0 | 6.3 | 6.4 | 0.5 |
| Blood | 7.9 | 9.0 | 8.3 | 8.4 | 0.3 |
| Nasal Mucosa | 1.0 | 7.0 | 3.6 | 3.4 | 2.6 |

Table 4: Descriptive statistics of Fragment Size (bp) for each tissue sample category collected (dried muscle, ear stored in Allflex [ET-All] or ethanol [ET-Eth], frozen retropharyngeal lymph nodes [RLNs], refrigerated RLNs, tongue, blood, and nasal mucosa) per 1-2 μL of eluted DNA for evaluation of requirements for use in a double digest restricted site associated DNA sequencing, high-density, and medium-density sequencing panels.

| **Sample** | **Min** | **Max** | **Mean** | **Median** | **StDev** |
| --- | --- | --- | --- | --- | --- |
| Dried Muscle | 13920 | 31367 | 18913 | 18231 | 4154 |
| ET-All | 19931 | 54598 | 42165 | 49233 | 11194 |
| ET-Eth | 20349 | 58563 | 35205 | 31445 | 10626 |
| Frozen RLN | 1406 | 24203 | 14478 | 13720 | 5508 |
| Refrigerated RLN | 11434 | 19295 | 14888 | 14030 | 2652 |
| Tongue | 14565 | 23504 | 18124 | 17562 | 2424 |
| Blood | 18089 | 44922 | 25630 | 23194 | 6901 |
| Nasal Mucosa | 137 | 17753 | 8393 | 10329 | 6761 |
